# Polypharmacy and the occurrence of potential drug–drug interactions among geriatric patients at the outpatient pharmacy department of a regional hospital in Durban, South Africa

**DOI:** 10.1186/s40545-021-00401-z

**Published:** 2022-01-04

**Authors:** Adetola Olaniyi Bojuwoye, Fatima Suleman, Velisha Ann Perumal-Pillay

**Affiliations:** Discipline of Pharmaceutical Sciences, University of KwaZulu-Natal, Private Bag X54001, Durban, 4000 South Africa

**Keywords:** Polypharmacy, Drug interactions, Geriatric, South Africa

## Abstract

**Background:**

Polypharmacy is the administration of an excessive number of medicines and a significant irrational medicine use practice. Little is known about this practice in South Africa. This study aimed to determine the level of polypharmacy and potential drug–drug interactions amongst the geriatric patient population in a facility in South Africa.

**Method:**

A cross-sectional retrospective prescription chart review for 250 geriatric patients was conducted at the outpatient pharmacy department of a regional hospital. Variables extracted included demographic information, diagnosis, type of prescriber contact, and polypharmacy. Potential drug–drug interactions were determined with web-based multi-drug interaction checkers.

**Results:**

The average (SD) number of diagnosed clinical problems was 3.54 ± 1.26, with hypertension, diabetes mellitus, and heart disease occurring most frequently. The level of polypharmacy was high with patients receiving an average (SD) of 12.13 ± 4.25 prescribed medicines from 3032 prescribed medicines. The level of polypharmacy was highest within the age categories, 60–64, and 70–74 years of age, respectively. The level of potential drug–drug interactions was also high with an average (SD) of 10.30 ± 7.48 from 2570 potential drug interactions. The majority of these interactions were moderate (72.5%) and pharmacodynamic (73.2%) by nature of the clinical severity of action and mechanism of action, respectively. Polypharmacy and type of prescriber contact were statistically significant contributors to the occurrence of potential drug–drug interactions, (*F* (2, 249) = 68.057, *p* < 0.05). However, in a multivariate analysis of variables to determine the strength of the association, polypharmacy was determined to be the strongest contributor to the occurrence of potential drug–drug interactions (*p* < 0.05) when compared with the type of prescriber contact (*p* value = 0.467). Therefore, irrespective of the type of prescriber contact, polypharmacy increases the potential for drug interactions among the sampled patient population.

**Conclusion:**

A comprehensive consideration of disease management guidelines, patient factors, and rational medicine review could be measurable strategies towards improving medicine use. This would also limit the occurrence of significant drug interactions among the geriatric patient population. A national study is required to determine if differences occur across hospitals and regions.

## Background

According to the World Health Organisation (WHO), inappropriate medicine use (e.g., polypharmacy) is a significant public health problem due to a potential for medicine-related harm [1, 2]. Polypharmacy is described as the administration of an excessive number of medications and a significant irrational medicine use practice [2, 3]. Furthermore, while polypharmacy can be considered beneficial in specific conditions and patient populations, among the older patient population with complex age-related deterioration in renal and hepatic physiological functions, decreased body volume, and reduced body mass, inappropriate polypharmacy increases the risk of negative outcomes, such as adverse drug reactions (ADRs) and drug–drug interactions (DDIs) [4]. These outcomes are influenced by patient factors (e.g., age, gender, and patient’s physiology) and medicine use properties, such as duration of combined therapy, and can produce insignificant to potentially harmful effects which can be deleterious to a patient’s overall health and well-being with negative consequences, such as medication-related hospitalisations as well as increased morbidity and mortality [5–7].

The geriatric population are 60 years of age and older [8], represent a significant portion of the global population (approximately 962 million in 2017) and are regarded as significant consumers of prescription medications [9, 10]. This is attributable to a plenitude of multimorbid, non-communicable, and chronic conditions prevalent among geriatrics and often require long-term clinical care and complex pharmacotherapy [8, 11, 12]. In a developing country, such as South Africa, medicine use among the aging patient population is substantially high with older persons, responsible for about 38% of all prescribed medicines in 2011 [13]. Therefore, with polypharmacy appearing empirically unavoidable among the geriatric patient population [14], an improved understanding of medicine use practices among geriatrics is crucial in developing countries towards reducing medicine-related harm and associated adverse health outcomes.

Medicine use among the older patient population can be measured by the number of prescribed medicines received. Accordingly, the level of clinically relevant ADRs due to polypharmacy or complex polytherapy among a population of patients would also provide an estimation of medicine use or the burden of medicine-related morbidity and mortality on a healthcare system [15]. Research literature of studies conducted mostly in developed countries have reported significant associations between polypharmacy, drug interactions, hospitalisations as well as increased healthcare costs [16]. However, in developing countries, such as South Africa, with varying differences in age-groups, disease prevalence, and healthcare systems compared with developed countries [15, 17], polypharmacy and the impact of ADRs on healthcare services has not been studied extensively, thus, the negative consequences of polypharmacy may go undetected. Therefore, this study focused on determining the level of polypharmacy and potential drug–drug interactions (PDDIs) present among the ambulatory geriatric patient population presenting to a typical healthcare facility in South Africa. This is crucial for understanding the clinical benefits and potential harm of polypharmacy which may go undetected in the management of older patients while improving current research information on medicine use among the older patient population in South Africa. Furthermore, the significant role of pharmacists in clinical care and patient management is highlighted towards the improvement of rational medicine use in the context of potentially inappropriate polypharmacy and the negative effects of drug interactions in public healthcare institutions in South Africa. The influence of factors such as the number of co-morbidities, type of prescriber contact, and gender on polypharmacy and the occurrence of potential drug-related harm is also reviewed.

## Methods

### Study design

This research study involved a cross-sectional, retrospective review of prescription charts for geriatric patients, 60 years and older, received at the out-patient pharmacy department of a public healthcare facility over two weeks in February 2019.

### Study location

This study was conducted at a regional or level two public healthcare facility, in eThekwini, KwaZulu-Natal, South Africa. The hospital is a 350-bed facility with an average catchment population of approximately 34,000 patients [18] inclusive of up-referrals from the community and primary healthcare clinics for advanced treatment and down-referrals from tertiary level hospitals for the continuation of care and medication management.

### Study population

The out-patient pharmacy department of this hospital caters to approximately 400 to 500 patients a day according to available monthly pharmacy statistics which was reviewed before the initiation of this research study. Prescription charts of geriatric patients were selected at random for retrospective review as discussed below.

### Sample size

The sample size required for this study was determined using a free sample-size calculator available on the webpage, www.raosoft.com. The sample size calculator allows for the generation of a sample population by reducing selection bias through a 5% margin of error, at a 95% confidence interval, and a 50% response distribution which reduces skewness of the sample size and allows for the calculation of the largest sample size possible.

An approximate population sample of 120 geriatric patients, 60 years and older, on one of the busiest days, was used to estimate the average number of ambulatory geriatric patients (determined to be 60 per day) presenting to the out-patient pharmacy of the hospital. This was done to limit selection bias and an over-estimation of population size, since no official statistics existed per age-category. A sample population of 250 geriatric prescription charts was subsequently calculated using the online calculator and was considered sufficient for a cross-sectional analysis when compared to similar studies on polypharmacy and the occurrence of PDDIs [7, 17, 19, 20].

Polypharmacy was defined quantitatively for this research study as the concurrent use of 3 or more prescribed medicines, according to literature reviews on polypharmacy which determined that the quantification of polypharmacy is a widely accepted concept in research and clinical practice [10] in addition to WHO’s core medicine use indicators which evaluates prescribing practices by an average number of prescribed medicines per patient and by encounter [3]. Furthermore, prescription charts containing multiple medications (3 or more) can be concurrently analysed sufficiently for the occurrence of PDDIs using any available web-based multi-drug interaction checker [21–23].

#### Inclusion/exclusion criteria

- Inclusion criteria
  Prescription charts of ambulatory geriatric patients (60 years and older).
  Prescription charts with a minimum of 3 medications.
- Exclusion criteria
  i. General exclusion criteria.
    Prescription charts with incomplete data and information.
    Prescription charts of patients 59 years and younger.
    Prescription charts with primarily creams, ointments, and compounded items. These items were included in the analysis for polypharmacy if prescribed with other pharmaceutical formulations.
    Prescription charts with antiretroviral (ARV) medication were excluded as access to ARV prescription charts collection was restricted.
  ii. Exclusion criteria for analysing potential drug interactions.
    The analysis of prescribed medications for potential drug–drug interactions included only solid oral dosage forms as other dosage forms (e.g., injectables) did not fall within the scope of this research study.
    Therefore, the following items were excluded from drug interaction analysis:
    Insulin
    Supplements such as multivitamins, thiamine, and pyridoxine (except magnesium chloride and calcium carbonate)
    Specific liquids, i.e., liquid paraffin and Shohl’s solution.
    Creams and Ointments—Excluded from drug interaction analysis as these items typically produce therapeutic effects localised at the application site. Furthermore, while absorption is expected, the systemic effect is minimal except if used for a long period [24]

### Research ethics and permissions

The study received ethics approvals from the University of KwaZulu-Natal Biomedical Research Ethics Committee (BREC REF: BE651/18); the KZN Department of Health (KZ_201811_034) as well as the subject hospital, where data were collected.

### Data collection tool

According to the WHO in collaboration with the International Network for Rational Drug Use (INRUD), medicine use indicators are measures of performance relating to the appropriate use of medicines by health care providers [2, 3]. These medicine use indicators are objective core tools that can be used quickly and efficiently to identify potential medicine use problems in addition to prioritizing solutions towards correcting a problem in patient management [3]. Furthermore, data on prescribing indicators such as the average number of medicines per encounter can be retrospectively collected and analysed as required for a cross-sectional study on prescribing patterns, polypharmacy, and the rational use of essential medicines [2, 3].

Therefore, the use of a WHO/INRUD prescriber and detailed encounters proforma data collection sheet for the analysis of core medicine use indicators such as polypharmacy satisfies the requirements of this research study and when modified, allows for the extraction of information sufficient for the inclusion and analysis of potential drug–drug interactions.

### Data collection process

Data collection occurred over 2 weeks between the 4th and the 15th of February 2019. A simple random sampling of prescription charts that satisfied the requirements of the inclusion criteria was conducted on defined days with three alternate days in the first week (Monday, Wednesday, and Friday) and two alternate days (Tuesday and Thursday) in the second week of data collection. Alternate prescription charts were selected at different times on the selected days.

Thereafter, selected prescription charts were scanned and anonymised by removing patient and prescriber information. Each scanned prescription chart was assigned a code and number for data extraction and stored on a dedicated, single access storage device with a password known only to the researcher. The scanned prescription charts will be deleted on completion of the study according to the ethical requirements of data management and the rules of BREC—University of KwaZulu-Natal.

Essential data and information from selected prescription charts were obtained with a modified WHO/INRUD prescriber indicator and detailed encounters form—described above. This included demographic information such as age and gender in addition to data on the type of prescriber contact, number of diagnosed clinical problems, prescribed medicines as well as the results of drug interaction analysis for potential drug–drug interactions, PDDIs.

### Data analysis

The diagnosed clinical problems (acute and chronic) identified during the review of prescriptions charts were analysed with *WHO’s International Classification of diseases (ICD–11)* for simplification of data, with theoretically similar clinical problems combined under a common disease category. ICDs are often used in clinical care and research to define diseases, monitor outcomes, and allocate essential resources where required [25].

The level of polypharmacy for this research study was calculated by a within-age comparison. This was estimated as a percentage by dividing the number of people with polypharmacy in each defined age-category by the total number of people in the representative sample.

#### Data analysis—potential drug–drug interactions

The prescribed medicines extracted during the review of prescription charts that satisfied the criteria for drug interaction assessments were analysed for the occurrence of PDDIs with web-based multi-drug interaction checkers, Epocrates online, and Medscape drug reference interaction checker. The result of the analysis was recorded on the modified data extraction proforma used for this research study.

Epocrates online and Medscape drug references are one of many freely available drug information databases widely used in clinical research and practice [21]. These online or web-based drug information databases have sufficient sensitivity, i.e., the ability to detect clinically relevant interactions and specificity, i.e., to ignore clinically unimportant and irrelevant drug interactions, to be used as reliable clinical support tools in clinical care settings by providing dependable medicine information and medicine use recommendations which are essential for the improvement of clinical care and pharmacotherapy [17, 22].

The outcome of drug interaction analysis for this research study are classified as potential drug interactions. This is because actual drug interactions cannot be determined with retrospective chart reviews. Furthermore, actual drug interactions can only be determined with smaller and controlled research studies inclusive of complete patient information, diagnosis, and complete medicine use history [26, 27].

#### Data and statistical analysis

All extracted data and information on completed proformas were transferred onto a computerized spreadsheet using Google® sheets for ease of analysis. Descriptive analysis for continuous and categorical variables was calculated using the software program, *Statistical Package for Social Sciences*, SPSS version 25. The result from the analysis of variables was expressed in mean and standard deviation for continuous variables (e.g., age), while categorical variables (e.g., gender) were expressed in percentages.

Potential associations or relationships between the predictor variables (i.e., polypharmacy, type of prescriber contact, and gender) and the outcome variable (i.e., the occurrence of PDDIs) were determined with the Chi-square test of associations. This is a non-parametric test that is often used to determine significant associations between variables reviewed in a research study. Furthermore, the Chi-square test of association provides relevant information regarding the association or independence of the reviewed variables and is easy to compute on the statistical software, SPSS [28]. The statistical software program has been validated for the evaluation of large amounts of data while providing outcome reports, such as summary statistics as well as descriptive relationships between variables.

Significant relationships identified using Chi-square tests of association were subsequently included in a multivariate analysis of variables. Multiple regression analysis is often used to determine the strength of the relationship between several predictor variables and the outcome [29]. Therefore, the relative influence of the predictor variables (i.e., polypharmacy, type of prescriber contact, and gender) on the outcome variable (i.e., the occurrence of potential drug–drug interactions) as well as the strength of the associations were assessed in this study.

The level of significance for statistical tests, the *p* value, was set at *p* < 0.05.

## Results

### Demographic characteristics

Prescription charts of 250 geriatric patients, 60 years and older, were reviewed for this research study. The demographic information obtained from the reviewed prescription charts included age, gender, and type of prescriber contact, i.e., primary patient contact with either a level 2 prescriber (regional hospital doctor) or a level 3 hospital doctor (specialist prescriber). Other demographic information such as educational status, employment history, income, and patient’s race could not be ascertained or verified as the research study was retrospective and did not include patient contact or interviews.

The average (SD) age in years of sampled geriatric patients was 69.72 ± 7.22. The majority of the sample population were female, 67.6% (*n* = 169), with males comprising 32.4% (*n* = 81). This resulted in a female to male ratio of 2:1 and a potential limitation for the differences in results between females and males observed in this research study.

Furthermore, with regards to the type of prescriber contact and reviewed patient population, 74.8% (*n* = 187) of reviewed prescription charts contained medications ordered primarily by level 2 prescribers, while 25.2% (*n* = 63) of reviewed prescription charts contained medications ordered by level 3 or specialist prescribers.

The demographic characteristics of the study population are presented in Table 1.

**Table 1:** Summary of demographics and clinical variables

Demographics	Frequency (*n* = 250)
Gender	
Female	169 (67.6%)
Male	81 (32.4%)
Age (years)	
60–64	77 (30.8%)
65–69	48 (19.2%)
70–74	64 (25.6%)
75–79	34 (13.6%)
80–84	16 (6.4%)
> 85	11 (4.4%)

Clinical variables	Frequency (*n* = 250)
Diagnosed clinical problems	
1	10 (4.0%)
2	38 (15.2%)
3	81 (32.4%)
4	69 (27.6%)
> 5	52 (20.8%)
Number of prescribed medicines	
3–6	26 (10.4%)
7–10	62 (24.8%)
11–14	90 (36.0%)
15–18	50 (20.0%)
> 19	22 (8.8%)
Number of potential drug–drug interactions	
0–10	146 (58.4%)
11–20	80 (32.4%)
21–30	19 (7.6%)
31–40	4 (1.6%)
> 41	1 (0.4%)

### Prescription chart variables

The variables extracted from reviewed prescription charts included the following: the number of diagnosed clinical problems (acute and chronic), the number of prescribed medications, and the number of potential drug–drug interactions identified according to the clinical severity and mechanism of actions.

#### The number of diagnosed clinical problems

The prescription charts of sampled geriatric patients, 60 years and older, produced 844 diagnosed clinical problems with an average (SD) of 3.54 ± 1.26. Hypertension (*n* = 223; 25.2%), diabetes mellitus (*n* = 146; 16.5%) and dyslipidemia (*n* = 97; 10.9%), accounted for the most diagnosed clinical problems.

The data presented in Table 2 represents the total number of diagnosed clinical problems identified in this research study per age category and gender.

**Table 2:** Total number of diagnosed clinical problems per age category and gender

Variable	Frequency—diagnosed clinical problems^a^		Total	Percentage
Age^b^	Female	Male	*n* = 884	%
60–64	178	94	272	30.8
65–69	93	64	157	17.8
70–74	169	77	246	27.8
75–79	80	42	122	13.8
80–84	36	16	52	5.8
> 85	29	6	35	4.0

^a^Frequency counts by age and gender

^b^Age in Categories in years

#### The number of prescribed medicines

**Fig. 1:**
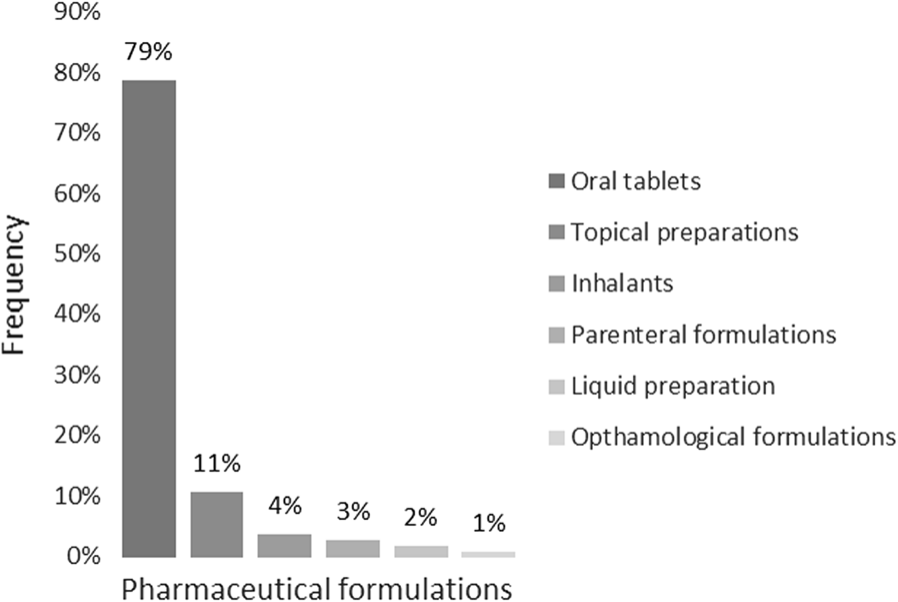
Prescribed medicines by pharmaceutical formulation

One hundred and thirty-six (136) medicines classified by pharmaceutical preparation was prescribed for the geriatric patient population reviewed for this research study. These medicines consisted of varied combinations of oral tablets, topical preparations, inhalants (e.g., nasal sprays and nebulizers), parenteral formulations (e.g., Insulin), liquid preparation, and ophthalmological formulations. Figure 1 represents the distribution of prescribed medicines by pharmaceutical formulation.

The total number of prescribed medicines for this research study was 3032 with an average (SD) of 12.13 ± 4.25. Female geriatric patients were prescribed an average (SD) of 12.45 ± 4.51, while the male geriatric patients received on average (SD) 11.46 ± 3.58 prescribed medicines.

#### The number of potential drug–drug interactions

According to the data extracted from reviewed prescription charts, 63.9% (*n* = 87) of prescribed medicines classified by pharmaceutical formulations were oral tablet formulations, equivalent to 78.9% (*n* = 2394) of all prescribed medicines. The oral tablet formulations were subsequently analysed for the occurrence of potential drug–drug interactions, PDDIs, using the free online multidrug interaction checkers, Epocrates, for clinical severity of action due to a large medicine reference database [30]. These interactions are described as minor (caution advised), moderate (monitor or modify treatment), major (avoid or use alternative), and contraindicated interactions [30, 31]. Medscape drug interaction checker was used for the analysis of the mechanism of action, i.e., pharmacodynamic, pharmacokinetic, and unknown interactions, due to a definite indication for the type of mechanism of action compared to Epocrates.

The analysis for potential drug interactions per prescription chart produced 2570 PDDIs with an average (SD) of 10.30 ± 7.48 per patient from 95.4% (*n* = 83) of oral tablet formulations. One liquid preparation was included in the analysis due to the active ingredient in the preparation, morphine sulphate.

**Fig. 2:**
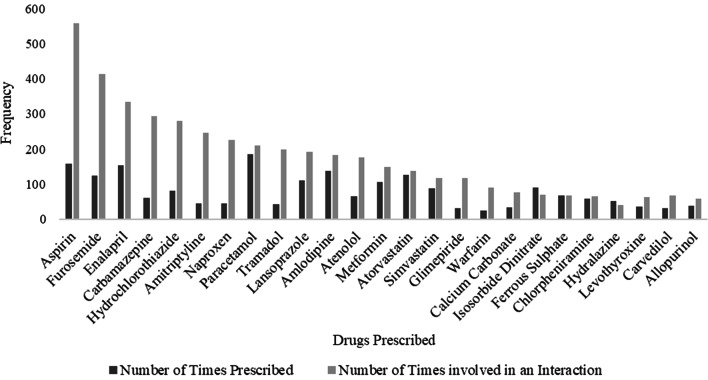
Prescribed oral tablet formulations and the number of involvements in PDDIs

The most-prescribed oral tablet formulation for this research study was paracetamol, appearing on 73.6% (*n* = 184) of prescription charts with a frequency of 210 involvements in PDDIs. However, aspirin was responsible for the most PDDIs (*n* = 559) although the medicine was prescribed 157 times (62.8%) compared to paracetamol, 184 times (73.6%) (Fig. 2).

#### Potential drug interactions by severity and mechanism of action

The total number of PDDIs identified in this research study was 2570. Potential interactions by clinical severity of action produced minor (*n* = 350; 13.6%), moderate (*n* = 1863; 72.5%), major (*n* = 349; 13.6%) and contraindicated interactions (*n* = 8; 0.3%), while PDDIs by mechanism of action produced pharmacokinetic (*n* = 604; 23.5%), pharmacodynamic (*n* = 1882; 73.2%) and unknown interactions (*n* = 84; 3.3%).

A summary of interacting drug pairs identified in this research study with a probability of clinical significance (i.e., major interactions, which is described as drug interactions that require routine clinical intervention or therapeutic dose monitoring to minimize or prevent adverse effects that may be fatal or detrimental to a patient's overall health and contraindicated interactions, which is described as drug interactions which produce clinically significant drug interactions and are discouraged in clinical practice due to the potential for severe adverse reactions) is presented in Table 3.

**Table 3:** Summary of interacting drug pairs with a potential for clinical significance—major and contraindicated drug interactions

Interacting drug pairs		Mechanism of action	Frequency	Percentage
Major drug interactions		PD/PK/UNK	*n* = 349	%
Paracetamol	Carbamazepine	PK	50	14.3
Amlodipine	Simvastatin	PK	43	12.3
Ferrous sulphate	Lansoprazole	PK	33	9.5
Carbamazepine	Simvastatin	PK	26	7.4
Allopurinol	Aspirin	PD	24	6.9
Aspirin	Naproxen	PD	23	6.6
Lansoprazole	Naproxen	PD	20	5.7
Amitriptyline	Tramadol	PD	17	4.9
Aspirin	Clopidogrel	PD	13	3.7
Carbamazepine	Tramadol	PK	13	3.7
Atenolol	Naproxen	PD	12	3.4
Hydralazine	Tramadol	PD	10	2.9
Fluconazole	Simvastatin	PK	8	2.3
Aspirin	Warfarin	PD	5	1.4

Contraindicated drug interactions		PD/PK/UNK	*n* = 8	%
Amitriptyline	Potassium Chloride	PK	1	12.5
Desmopressin	Hydrocortisone	PD	1	12.5
Tamoxifen	Warfarin	UNK	1	12.5
Fluconazole	Tramadol	PK	5	62.5

*PD* pharmacodynamic, *PK* pharmacokinetic, *UNK* unknown

### Reviewed associations between variables

#### Polypharmacy and geriatric patients

The level of polypharmacy for this research study was determined by the average number of medicines prescribed within a particular age category. Geriatric patients within the age-categories, 60–64 (12.39; *n* = 954; 31.5%) and 70–74 (11.97; *n* = 766; 25.2%) years of age had the highest levels of polypharmacy compared to the other age-categories reviewed in this research study. The level of polypharmacy was also determined to be highest among the female geriatric population with females receiving an average of 12.45 prescribed medicines (*n* = 2104; 69.4%) compared to males, 11.45 (928; 30.6%).

A chi-square analysis (*p* < 0.05) between the variables, gender and polypharmacy was considered statistically significant, χ^2^ (1, *n* = 250) = 6.177, *p* = 0.013. Therefore, a significant relationship was confirmed between gender (females) and the occurrence of polypharmacy according to the results of this research study.

#### The number of prescribed medications and potential drug–drug interactions

Two hundred and forty-one (241) prescription charts reviewed produced at least one PDDI with an average of 10.30 (SD ± 7.48) potential interactions.

A chi-square statistical analysis (*p* < 0.05) to determine a potential relationship between the number of prescribed medicines and the occurrence of PDDIs was determined to be statistically significant, χ^2^ (1, *n* = 250) = 14.42, *p* < 0.05, therefore, confirming a positive relationship between the number of prescribed medicines and the occurrence of PDDIs according to the results of this research study.

#### The number of prescribed medications, potential drug–drug interactions, and type of prescriber contact

The prescription charts sampled for this research study with medications ordered primarily by level 2 prescribers (*n* = 187) produced 2193 prescribed medicines with an average (SD) of 11.44 ± 4.08 and 1771 PDDIs with an average (SD) of 9.19 ± 7.05. Geriatric prescription charts with medications ordered by specialist or level 3 prescribers (*n* = 63) produced 893 prescribed medicines with an average (SD) of 14.17 ± 4.14 and 853 PDDIs with an average of 13.57 ± 7.82.

A chi-square analysis (*p* < 0.05) to determine potential associations between prescriber contact and the number of prescribed medicines, χ^2^ (1, *n* = 250) = 11.62, *p* < 0.05 as well as prescriber contact and the number of PDDIs, χ^2^ (1, n = 250) = 5.99, *p* < 0.014, was statistically significant. Therefore, a positive bivariate relationship was confirmed between the type of prescriber contact (i.e., level 3 or specialist prescriber) and the number of prescribed medicines as well as the occurrence of PDDIs.

#### Multivariate test of associations between variables

A summary of significant results from the chi-square test of associations (chi-square, *p* < 0.05) between the reviewed variables is presented in Table 4.

**Table 4:** Summary of results from chi-square tests to determine significant relationships

Gender and polypharmacy			
Variable	Number of prescribed medicines^a,b^		*p* value (*p* < 0.05)
Gender	3–15	> 15	0.013*
Female	44.8%	22.8%	
Male	26.4%	6.0%	

The number of diagnosed clinical problems and type of prescriber contact			
Variable	Number of diagnosed clinical problems^a^		*p* value (*p* < 0.05)
Prescriber contact	0–4	5–8	0.013*
Level 2	62.0%	12.8%	
Level 3	17.2%	8.0%	

Polypharmacy and type of prescriber contact				
Variable	Number of prescribed medicines^a^			*p* value (*p* < 0.05)
Prescriber Contact	3–8	9–14	> 15	0.01*
Level 2	17.6%	40.0%	17.2	
Level 3	2.8%	10.8%	11.6%	

Polypharmacy and potential drug–drug interactions			
Variable	Number of prescribed medicines^a,b^		*p* value (*p* < 0.05)
Number of PDDIs^a^	3–14	> 15	< 0.05*
0–20	70.0%	20.4%	
> 21	1.2%	8.4%	

Type of prescriber contact and potential drug–drug interactions			
Variable	Number of PDDIs^a^		*p* value (*p* < 0.05)
Prescriber Contact	0–20	> 21	0.014*
Level 2	69.6%	5.2%	
Level 3	20.8%	4.4%	

^a^Frequency counts in percentages

^b^The number of prescribed medicines was collapsed into two categories, because smaller observations were observed for the categories 3–8 and 9–14 of the number of prescribed medicines when analysed against the number of PDDIs (> 21). This was also to satisfy the assumption of chi-square tests of association which suggests that at least one observation is present in each cell of the table. If not, categories can be collapsed to form meaningful categories [28, 29]

*Statistically Significant results

According to the results of the statistical tests presented in Table 4, significant associations were confirmed between variables; the number of prescribed medicines, type of prescriber contact, and the outcome variable, i.e., the occurrence of PDDIs. Therefore, to determine the existence of a significant multivariate relationship between the dependent variables, i.e., the type of prescriber contact and the number of prescribed medicines towards the outcome variable, i.e., the occurrence of PDDIs among the sampled geriatric patient population, a multiple regression analysis was conducted and the results are presented in Table 5.

**Table 5:** Model summary from multiple regression analysis of variables

Variables^a^	Sum of Squares	Df^b^	Mean Square	F	*p* value
Regression	48.381	2	24.191	68.057	0.000^c^
Residual	87.795	247	0.355		
Total	136.176	249			

^a^Polypharmacy, Prescriber Contact and outcome variable: Potential drug interactions

^b^Degrees of freedom

^c^*p* < 0.05

The results in Table 5 show that the number of prescribed medicines together with the type of prescriber contact significantly predicts the occurrence of PDDIs, *F* (2, 249) = 68.057, *p* < 0.05, therefore, confirming a mutual multivariate association between polypharmacy, type of prescriber contact, and occurrence of PDDIs.

However, further analysis to determine the relative strength or contribution towards this mutual association as co-variables, showed that the number of prescribed medicines or polypharmacy was the strongest contributor towards the occurrence of PDDIs with a standardized coefficient beta of 0.583 (statistically significant, *p* < 0.05). The type of prescriber contact was statistically insignificant (*p* value = 0.467), providing a weaker contribution as a co-variable towards the occurrence of a PDDI according to the results of this research study.

The summary of the analysis showing the relative contribution of the variables; the number of prescribed medicines (polypharmacy) and the type of prescriber contact towards the occurrence of PDDIs are presented in Table 6.

**Table 6:** Relative contribution of significant variables towards the occurrence of PDDIs

Variable	Standardized coefficients beta	*p* value
Potential drug–drug interactions^a^		0.05
Polypharmacy	0.583	**0.000**
Prescriber contact	0.039	0.467

^a^Outcome variable

## Discussion

Pharmacotherapy in patient care and disease management is essential for improving optimal health. However, the inappropriate use of multiple medicines poses a significant public health challenge due to adverse medicine-related events which can impact negatively on a patient’s overall health and well-being [1, 2]. Therefore, the primary objective of this research study was to determine the level of polypharmacy and potential drug interactions amongst geriatric patients receiving medicines at a typical healthcare facility in South Africa.

Potentially excessive polypharmacy was identified in this research study with the sampled geriatric patient population receiving on average 12.13 prescribed medicines per encounter reviewed. The level of polypharmacy was found to be higher among female geriatric patients as well as on medications ordered by specialists or level 3 prescribers. Consequently, the high level of polypharmacy identified in this research study produced a correspondingly high number of potentially significant drug interactions with the majority of the reviewed prescription charts (96.4%) producing at least one potential drug interaction. The inference drawn from these outcomes would indicate that the geriatric patient population of the reviewed healthcare facility are likely to be prescribed multiple medicines and complex pharmacotherapy which can result in potentially significant clinical interactions as well as medicine-related harm.

Accordingly, other studies have reported similar outcomes identified in this research study albeit a few exclusions. The results of a Brazilian study [32] for example reported a high number of prescribed medicines (7 or more) which was significant towards the occurrence of drug interactions. However, this outcome was specific for non-ambulatory and female cardiology patients who are 55 years of age and older. Likewise, the results of an Indian study investigating the prevalence of potentially inappropriate medication in elderly patients at a tertiary care teaching hospital [33] and a South African study investigating potential drug interactions in primary healthcare clinics [17] also reported a correlation between increasing polypharmacy and the occurrence of potential drug interactions. However, while gender was considered an insignificant factor in both studies, the influences of increasing aging and specialist prescribers were identified in the South African study as significant and contributory factors towards the occurrence of potential drug interactions. The varied differences between this current research study and other studies can perhaps be explained from the outcome of a literature review on polypharmacy among the elderly patient population [34]. This review suggested that while polypharmacy was a common phenomenon among the elderly with a prevalence rate of between 5 and 78%, the results from reviewed research studies on polypharmacy are often influenced by differences in the definition of polypharmacy, methodology, and the sample sizes of the reviewed studies. Furthermore, while this current research study described polypharmacy quantitatively as the prescribing of 3 or more medicines, it is noteworthy to indicate that majority of the patient population (97.6%) were prescribed 5 or more prescribed medications. This was attributed in some part to the prescribing guidelines used in public healthcare institutions which are generally disease-specific and advocate for intermediate outcomes, such as normalised blood pressure in hypertensive patients or optimal glycaemic control in diabetic patients [35], without a holistic review of a patient’s health status and medicine use properties of the prescribed medications.

Polypharmacy is relatively unavoidable among the older patient population due in part to a higher prevalence of co-morbid conditions among this patient population in addition to other healthcare-related factors, such as the use of disease-specific guidelines in patient care and disease management [19, 34]. Thus, a rational review of medicines prescribed to and used by the geriatric patient population is crucial towards identifying factors that contribute to polytherapy among the older patient population as well as mechanisms and tools for the reduction of adverse medicine-related events which impact negatively on healthy aging.

The research literature on polypharmacy has highlighted mechanisms and tools for improving medicine use. The use of prescribing practices such as deprescribing (i.e., a systematic review of prescribed medications either for reduction or discontinuation) [36], the incorporation of pharmacist recommendations, patient education on rational medicine use as well as the use of peer-reviewed evidence-based guidelines (e.g., Screening Tool to Alert Doctors to Right Treatment (START), Screening Tool of Older People’s Potential Inappropriate Prescriptions (STOPP) and Beers criteria) [36–39] have been identified as effective methods for improving medicine use amongst the older patient population. These tools could be considered for South Africa towards an improvement in patient outcomes related to polypharmacy as well as medicine-related harm which impact negatively on optimal health and healthy aging.

### Limitations

Polypharmacy was defined quantitatively for this research study as the prescribing of 3 or more medications. However, the suitability of the prescribed medicines per diagnosis was not reviewed. Therefore, while this study randomly selected and actively used geriatric prescription charts with 3 or more prescribed medicines, the results of this research study on the level of polypharmacy and by extension, the number of PDDIs could have been subject to bias, since polypharmacy was actively reviewed for this research study. Furthermore, the cross-sectional nature of this research study could be deemed a limiting factor as data was collected within a specific and limited period which may not have presented a true reflection on the nature of polypharmacy and the PDDIs present among the geriatric patient population at the facility reviewed for this research study.

The web-based or online multi-drug interaction checkers, i.e., Epocrates and Medscape, used in this research study for the analysis of PDDIs were determined to have sufficient sensitivity and specificity towards identifying a potential drug interaction. However, this study was retrospective in nature, therefore, the identified drug interactions could not be verified when significant contraindicated results were obtained. Furthermore, only 95.4% (*n* = 83 of 87) of eligible tablet formulations were included in the analysis for PDDIs, because the medicine catalogues for Medscape and Epocrates did not include the drugs; cinnarizine (antihistamine), hyoscine butyl bromide (antispasmodic), carbimazole (antithyroid agent) and bezafibrate (fibrate), which may have impacted on the results of potential drug interactions obtained in this research study.

### Recommendations

The results of this retrospective and quantitative research study suggest a high level of polypharmacy and PDDIs among the sampled patient population. However, while medicine use policies in South Africa, i.e., National drug policy (NDP) and Essential drug policy (EDP), encourage and promote the rational and efficient use of essential medicines [40], the occurrence of a high level of polypharmacy—an irrational medicine use practice [1], increases the risk for negative outcomes such as increases in healthcare costs due to unnecessary hospitalisations as well as a deterioration in health and well-being from clinically significant medicine interactions. Furthermore, the result of this research study would also appear to imply that sufficient protocols in medicine use and review are lacking, therefore, indicating a need for further research studies in prescribing practices in public healthcare and especially for the older patient population. In addition to these, the appropriateness of prescribed medicines, as well as the necessity of polypharmacy among this specific patient population, is worthy of further review. Supplementary research studies would also be required to determine the significance of clinically significant medicine interactions in older patients due to the impact of these interactions on medicine adherence, medicine safety as well as the health and well-being of this specific patient population.

## Conclusion

The population of older persons is steadily growing and currently represents a significant percentage of the global population [8, 13]. Therefore, this research study was crucial towards understanding the level of medicine use among a population of patients who often require long-term clinical care as well as complex pharmacotherapy [8, 11]. Furthermore, while this research study has identified a high level of polypharmacy in addition to a high occurrence of PDDIs in geriatric patients, a comprehensive review of disease management guidelines, prescribing protocols, patient factors, in addition to medicine utilisation and review protocols are also essential towards understanding and improving medicine use in geriatric patients. This process would further include broader research studies at other public healthcare facilities towards an understanding of the differences in prescribing and medicine use practices in geriatric patients countrywide. This could also be a potential role that pharmacists could play under the national health insurance models of care.

## Data Availability

The data extraction tools used during the current study are available from the corresponding author on reasonable request.

## Abbreviations

ADRs: Adverse drug reactions
ARVs: Antiretrovirals
BREC: Biomedical Research Ethics Committee
DDIs: Drug–drug interactions
DRPs: Drug-related problems
EDP: Essential Drugs Policy
ICD: International Classification of Diseases
INRUD: International Network for Rational Use of Drugs
NDP: National Drugs Policy
PDDIs: Potential drug–drug interactions
RDU: Rational drug use
SD: Standard deviation
SPSS: Statistical Package for the Social Sciences
WHO: World Health Organisation
